# Do Dental Resin Composites Accumulate More Oral Biofilms and Plaque than Amalgam and Glass Ionomer Materials?

**DOI:** 10.3390/ma9110888

**Published:** 2016-11-01

**Authors:** Ning Zhang, Mary A.S. Melo, Michael D. Weir, Mark A. Reynolds, Yuxing Bai, Hockin H.K. Xu

**Affiliations:** 1 Department of Orthodontics, School of Stomatology, Capital Medical University, Beijing 100050, China; dentistzhang112@163.com; 2 Department of Endodontics, Periodontics and Prosthodontics, School of Dentistry, University of Maryland, Baltimore, MD 21201, USA; mmelo@umaryland.edu (M.A.S.M.); MWeir@umaryland.edu (M.D.W.); MReynolds@umaryland.edu (M.A.R.); 3 Center for Stem Cell Biology & Regenerative Medicine, School of Medicine, University of Maryland, Baltimore, MD 21201, USA; 4 Marlene and Stewart Greenebaum Cancer Center, School of Medicine, University of Maryland, Baltimore, MD 21201, USA; 5 Department of Mechanical Engineering, University of Maryland, Baltimore, MD 21250, USA

**Keywords:** protein repellant, dental composite, human saliva microcosm biofilm, amalgam, glass ionomer, caries inhibition

## Abstract

A long-time drawback of dental composites is that they accumulate more biofilms and plaques than amalgam and glass ionomer restorative materials. It would be highly desirable to develop a new composite with reduced biofilm growth, while avoiding the non-esthetics of amalgam and low strength of glass ionomer. The objectives of this study were to: (1) develop a protein-repellent composite with reduced biofilms matching amalgam and glass ionomer for the first time; and (2) investigate their protein adsorption, biofilms, and mechanical properties. Five materials were tested: A new composite containing 3% of protein-repellent 2-methacryloyloxyethyl phosphorylcholine (MPC); the composite with 0% MPC as control; commercial composite control; dental amalgam; resin-modified glass ionomer (RMGI). A dental plaque microcosm biofilm model with human saliva as inoculum was used to investigate metabolic activity, colony-forming units (CFU), and lactic acid production. Composite with 3% MPC had flexural strength similar to those with 0% MPC and commercial composite control (*p* > 0.1), and much greater than RMGI (*p* < 0.05). Composite with 3% MPC had protein adsorption that was only 1/10 that of control composites (*p* < 0.05). Composite with 3% MPC had biofilm CFU and lactic acid much lower than control composites (*p* < 0.05). Biofilm growth, metabolic activity and lactic acid on the new composite with 3% MPC were reduced to the low level of amalgam and RMGI (*p* > 0.1). In conclusion, a new protein-repellent dental resin composite reduced oral biofilm growth and acid production to the low levels of non-esthetic amalgam and RMGI for the first time. The long-held conclusion that dental composites accumulate more biofilms than amalgam and glass ionomer is no longer true. The novel composite is promising to finally overcome the major biofilm-accumulation drawback of dental composites in order to reduce biofilm acids and secondary caries.

## 1. Introduction

Dental composites are increasingly popular as direct restorative materials for tooth cavity restorations [1,2,3,4]. The pace at which composites have been improved in the last 20 years is impressive, yielding better performance and longer service life in vivo [5,6,7,8]. Composites offer outstanding esthetics, suitable mechanical properties, and a conservative approach for cavity preparation. However, recurrent caries along the tooth-composite interfaces over time was identified as one of the predominant reasons for failure and replacement of composite restorations [2]. For example, a previous study showed that for class I posterior composite restorations, secondary caries was the cause for failure in 113 out of 129 cases of failure (88%), followed by restoration fracture [9]. Another study examined practice-based class II restorations for four to five years [10], and showed that secondary caries were a primary cause for failure (73.9%), followed by lost restorations (8.0%) and material fracture (5.3%). Oral bacteria, especially acidogenic bacteria, can produce acids that dissolve tooth minerals and degrade composite restorations [11]. Cariogenic bacterial adhesion and biofilm accumulation (dental plaque) on the surface of restorative materials were closely related to the development of secondary caries [12].

It has been reported over the years that resin composites had thicker biofilm formation and accumulation [13,14,15,16,17]. For example, “the percentage mutans streptococci of total CFU count in plaque was higher on composite (mean 13.7) and amalgam (mean 4.3) than on glass-ionomer (mean 1.1) restorations” [14]. Other studies observed that “resin composites inherently enhance bacterial growth” [15], and “there is a potential impact of composite resins on the ecology of microorganisms in the dental plaque biofilm”, due to increased biofilm buildup on composites [16]. The enhanced bacterial growth and plaque buildup on composites could lead to caries [15,17]. The reason that other direct-filling restorative materials such as glass ionomer cements and amalgam had less biofilms were related to the release of cariostatic agents such as fluoride ions, or Ag, Cu, and Zn ions [16]. However, glass ionomer cements are mechanically weak and cannot be used in large load-bearing restorations, and amalgam restorations are unesthetic and contain mercury, with declining applications [18]. Therefore, there is a need to develop a new composite not only with mechanical properties matching current composites, but also with reduced biofilms matching glass ionomer and amalgam materials.

Salivary proteins that adsorb onto enamel and dental material surfaces have been suggested as the prerequisite binding sites for initial oral bacteria attachment and biofilm development [19]. The colonization by cariogenic bacterium *Streptococcus mutans* (*S. mutans*) is thought to be initiated by attachment to salivary pellicle. Therefore, a protein-repellent strategy was recently developed to repel proteins from depositing onto resin surfaces, thereby repelling bacteria attachment [20]. The development of protein-repellent composite could potentially inhibit bacteria adhesion, leading to lower biofilm growth on restorations [21]. This approach in conjunction with other anti-caries strategies is promising to reduce secondary caries [22]. 2-methacryloyloxyethyl phosphorylcholine (MPC) is a methacrylate with a phospholipid polar group in the side chain leading to hydrophilic surfaces to reduce protein adsorption [23,24,25]. Recent studies incorporated MPC into dental adhesives and composites to reduce protein adsorption and bacteria attachment [26,27]. However, to date there has been no report on comparing the protein adsorption, bacteria attachment, and biofilm growth of the new protein-repellent composite with glass ionomer and amalgam materials. 

The objectives of this study were to investigate a protein-repellent composite containing MPC and compare its oral biofilm growth with glass ionomer and amalgam for the first time. It was hypothesized that: (1) Incorporation of MPC will greatly reduce protein adsorption on composite surface; (2) the new protein-repellent composite will have bacterial attachment, biofilm acid production, and biofilm CFU matching those of glass ionomer and amalgam controls; (3) the new protein-repellent composite will have strength and elastic modulus matching a commercial composite control without protein-repellent capability.

## 2. Materials and Methods

### 2.1. Fabrication of Protein-Repellent Composite

Bisphenol A glycidyl dimethacrylate (BisGMA) and triethylene glycol dimethacrylate (TEGDMA) (Esstech, Essington, PA, USA) were mixed at a mass ratio of 1:1, and rendered light-curable with 0.2% camphorquinone and 0.8% ethyl 4-*N*,*N*-dimethylaminobenzoate [27]. This is referred to as BT resin. MPC (Sigma-Aldrich, St. Louis, MO, USA), a methacrylate with a phospholipid polar group in the side chain, was used as the protein-repellent agent. The MPC powder was mixed with the BT resin at MPC/(BT + MPC) of 0% (control) and 10%, following previous studies [26,27]. A barium boroaluminosilicate glass of a mean particle size of 1.4 µm (Caulk/Dentsply, Milford, DE, USA) was silanized with 4% 3-methacryloxypropyltrimethoxysilane and 2% *n*-propylamine. The glass particles were mixed into each resin at 70% by mass. Because the resin mass fraction was 30%, the MPC mass fractions in the composite were: 0% (control), and 3%, respectively. 

A commercial composite (Heliomolar, Ivoclar, ON, Canada) was also tested. The fillers consisted of silica and ytterbium-trifluoride nano-fillers with particle sizes of 40–200 nm at a filler mass fraction of 66.7%. According to the manufacturer, Heliomolar is indicated for Class I and Class II restorations as well as Classes III–V restorations. 

In addition, a resin-modified glass ionomer cement (RMGI) (Vitremer, 3M, St. Paul, MN, USA) also served as a control. Vitremer consisted of fluoroaluminosilicate glass, and a light-sensitive, aqueous polyalkenoic acid. Indications include Class III, V and root-caries restoration, Class I and II in primary teeth, and core-buildup. A powder/liquid mass ratio of 2.5:1 was used according to the manufacturer. Furthermore, a dispersed-phase amalgam (Coutour, precapsulated admixed silver amalgam, Kerr, Orange, CA, USA) was also tested as a control. It consisted of 30%–60% mercury, 10%–30% silver, and 10%–30% copper, with 70% spherical and 30% lathe-cut particles. The following five materials were tested:

(1) 30% BT resin + 70% glass filler (referred to as “Composite with 0% MPC”);
(2) 27% BT resin + 3% MPC + 70% glass filler (“Composite with 3% MPC”);
(3) Commercial control composite Heliomolar (“Commercial composite control”);
(4) Resin-modified glass ionomer Vitremer (“RMGI”);
(5) Amalgam Coutour (“Amalgam”).

### 2.2. Mechanical Properties

Rectangular molds of 2 × 2 × 25 mm^3^ were used to make specimens for mechanical testing. For groups 1–4, each paste was placed into the molds. Each specimen was covered with mylar strips and photo-cured (Triad 2000, Dentsply, York, PA, USA) for 1 min on each open side. For group 5, each amalgam capsule was mixed using an amalgamator (Silamat S3, Ivoclar, Amherst, NY, USA) at 4500 oscillations/min for 7 s. After trituration, the mixed amalgam was condensed into the mold. All specimens were incubated for 24 h at 37 °C and then demolded. The specimens were immersed in distilled water at 37 °C for 24 h and then fractured in three-point flexure with a 10-mm span at a crosshead-speed of 1 mm/min on a computer-controlled Universal Testing Machine (5500R, MTS, Cary, NC, USA). Flexural strength (*S*) was calculated as: *S* = 3*P*_max_*L*/(2*bh*^2^), where *P* is the fracture load, *L* is span, *b* is specimen width, and *h* is thickness. Elastic modulus (*E*) was calculated as: *E* = (*P*/*d*)(*L*^3^/(4*bh*^3^)), where load *P* divided by displacement *d* is the slope in the linear elastic region. Specimens were fractured within a few minutes after being taken out of water [27].

### 2.3. Characterization of Protein Adsorption

For protein adsorption and biofilm experiments, disk molds of 9 mm in diameter and 2 mm in thickness were used to make the specimens [26,27], while following the same specimen fabrication procedures as described above. The specimens were immersed in distilled water at 37 °C for 24 h. Then the amount of protein adsorbed on composite disks was determined by the micro bicinchoninic acid (BCA) method [26,27]. Each disk was immersed in phosphate buffered saline (PBS) for 2 h. The disks then were immersed in bovine serum albumin (BSA) (Sigma-Aldrich) solutions at 37 °C for 2 h. The protein solutions contained BSA at a concentration of 4.5 g/L following previous studies [21,27]. The disks then were rinsed with fresh PBS by stirring at a speed of 300 rpm for 5 min (Bellco Glass, Vineland, NJ, USA), immersed in sodium dodecyl sulfate (SDS) 1% in PBS, and sonication at room temperature for 20 min to completely detach the BSA adsorbed onto the disk. A protein analysis kit (Fisher Scientific, Pittsburgh, PA, USA) was used to determine the BSA concentration in the SDS solution. Briefly, 25 µL of the SDS solution was mixed with 200 µL of the BCA working reagent in a 96-well plate, which was incubated at 60 °C for 30 min. Then the 96-well plate was cooled down to room temperature and the absorbance at 562 nm was measured via a microplate reader (SpectraMax M5, Molecular Devices, Sunnyvale, CA, USA). Standard curves were prepared using the BSA standard. From the concentration of protein, the amount of protein adsorbed on the composite disk surface was calculated [21,27].

### 2.4. Saliva Collection for Biofilm Inoculum

A dental plaque microcosm model was used following previous studies [28,29,30]. Saliva is ideal for growing dental plaque microcosm biofilms in vitro, with the advantage of maintaining much of the complexity and heterogeneity of the dental plaque in vivo. The saliva for biofilm inoculums was collected from 10 healthy adult donors having natural dentition without active caries or periopathology, and without the use of antibiotics within the last three months, following previous studies [28,29,30]. The donors did not brush teeth for 24 h and abstained from food and drink intake for 2 h prior to donating saliva. Stimulated saliva was collected during parafilm chewing and was kept on ice. An equal volume of saliva from each of the 10 donors was combined to form the saliva sample. The saliva was diluted in sterile glycerol to a concentration of 70%, and stored at −80 °C [28,29,30].

### 2.5. Dental Plaque Microcosm Biofilm Formation and Live/Dead Assay

The saliva-glycerol stock was added, with 1:50 final dilution, into the growth medium as inoculum [28,29,30]. The growth medium contained mucin (type II, porcine, gastric) at a concentration of 2.5 g/L; bacteriological peptone, 2.0 g/L; tryptone, 2.0 g/L; yeast extract, 1.0 g/L; NaCl, 0.35 g/L, KCl, 0.2 g/L; CaCl_2_, 0.2 g/L; cysteine hydrochloride, 0.1 g/L; hemin, 0.001 g/L; vitamin K1, 0.0002 g/L, at pH 7. The disks were sterilized in ethylene oxide (Anprolene AN 74i, Andersen, Haw River, NC, USA). 1.5 mL of inoculum was added to each well of 24-well plates with a disk, and incubated at 37 °C in 5% CO_2_ for 8 h. Then, the disks were transferred to new 24-well plates filled with fresh medium. After 16 h, disks were transferred to new 24-well plates with fresh medium and incubated for 24 h. This totaled 48 h of incubation, which was adequate to form microcosm biofilms as shown previously [29,31].

For live/dead assay, disks with two-day biofilms were washed with PBS and stained using the BacLight live/dead kit (Molecular Probes, Eugene, OR, USA). Live bacteria were stained with Syto 9 to produce a green fluorescence. Bacteria with compromised membranes were stained with propidium iodide to produce a red fluorescence. The stained disks were examined using an inverted epifluorescence microscope (TE2000-S, Nikon, Melville, NY, USA).

### 2.6. MTT Metabolic Assay

Disks with two-day biofilms were transferred to a new 24-well plate for the MTT (3-(4,5-Dimethylthiazol-2-yl)-2,5-diphenyltetrazolium bromide) assay [31]. MTT is a colorimetric assay that measures the enzymatic reduction of MTT, a yellow tetrazole, to formazan. A total of 1 mL of MTT was added to each well and incubated for 1 h. Disks were transferred to a new 24-well plate, and 1 mL of dimethyl sulfoxide (DMSO) was added to solubilize the formazan crystals. The plate was incubated for 20 min with gentle mixing at room temperature in the dark. Then, 200 µL of the DMSO solution from each well was collected, and its absorbance at 540 nm was measured via the microplate reader (SpectraMax M5). A higher absorbance is related to a higher formazan concentration, which indicates a higher metabolic activity in the biofilm on the disk [32].

### 2.7. Lactic Acid Production

The disks with two-day biofilms were rinsed with cysteine peptone water (CPW) to remove loose bacteria [31,32,33,34,35]. The disks were transferred to 24-well plates and 1.5 mL of buffered-peptone water (BPW) supplemented with 0.2% sucrose. The disks were incubated at 37 °C in 5% CO_2_ for 3 h to allow the biofilms to produce acid. The BPW solutions were then stored for lactate analysis. Lactate concentrations in the BPW solutions were determined using an enzymatic (lactate dehydrogenase) method [31,32,33,34,35]. The microplate reader was used to measure the absorbance at 340 nm for the collected BPW solutions. Standard curves were prepared using a lactic acid standard (Supelco, Bellefonte, PA, USA) [31,32,33,34,35].

### 2.8. Biofilm Colony-Forming Unit (CFU) Counts

Disks with two-day biofilms were transferred into tubes with 2 mL CPW, and the biofilms were harvested by sonication and vortexing (Fisher, Pittsburgh, PA, USA) [31,32,33,34,35]. Three types of agar plates were used to measure the CFU to assess microorganism viability. First, tryptic soy blood agar culture plates were used to determine total microorganisms. Second, mitis salivarius agar (MSA) culture plates containing 15% sucrose were used to determine total streptococci. This is because MSA contains selective agents including crystal violet, potassium tellurite, and trypan blue, which inhibit most Gram-negative bacilli and most Gram-positive bacteria except streptococci, thus enabling the streptococci to grow. Third, cariogenic mutans streptococci are known to be resistant to bacitracin, and this property is used to isolate mutans streptococci from the highly heterogeneous oral microflora. Therefore, MSA agar culture plates plus 0.2 units of bacitracin per mL was used to determine mutans streptococci. The bacterial suspensions were serially diluted, spread onto agar plates and incubated at 37 °C in 5% CO_2_ for 24 h. The number of colonies that grew was counted and used, along with the dilution factor, to calculate the CFU counts on each disk [31,32,33,34,35].

### 2.9. Statistical Analysis

One-way and two-way analyses of variance (ANOVA) were performed to detect the significant effects of the variables. Tukey’s multiple comparison test was used to compare the data at a *p* value of 0.05.

## 3. Results

**Figure 1:**
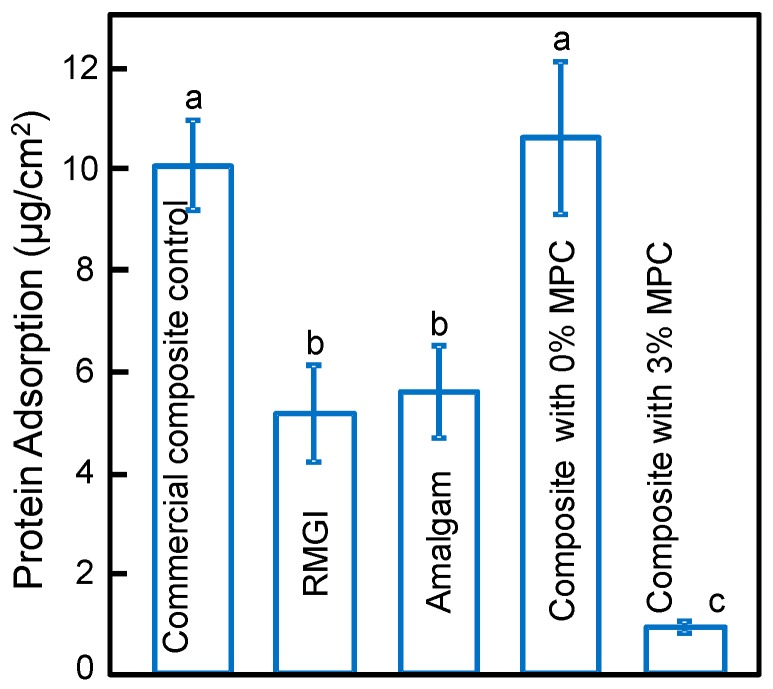
Protein adsorption onto disk surfaces (mean ± sd; *n* = 6). Incorporation of 3% MPC into the composite significantly decreased the amount of protein adsorption compared to that at 0% MPC and the commercial composite control (*p* < 0.05). Dissimilar letters indicate values that are significantly different from each other (*p* < 0.05).

The results on protein adsorption on disks are plotted in Figure 1 (mean ± sd; *n* = 6). RMGI and Amalgam had significantly less protein adsorption than the commercial composite and the control composite with 0% MPC (*p* < 0.05). Incorporation of 3% MPC into the composite greatly decreased the amount of protein adsorption, compared to control composite with 0% MPC and the commercial composite (*p* < 0.05).

**Figure 2:**
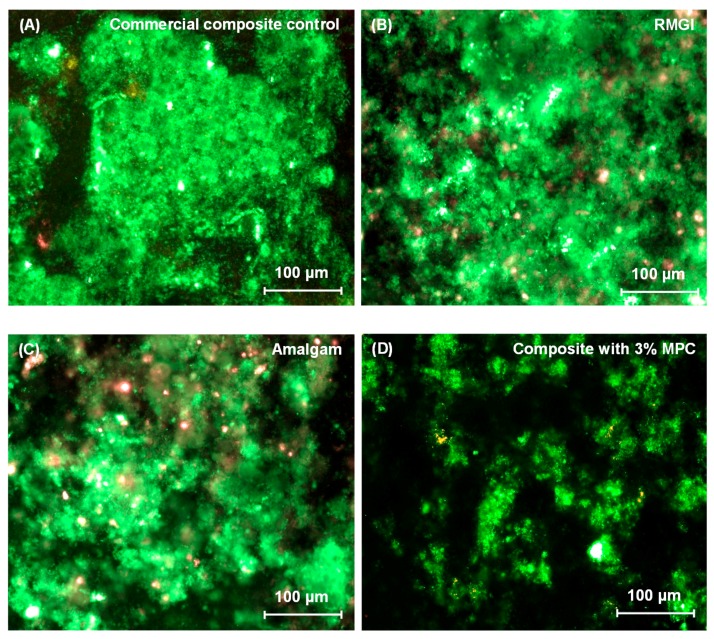
Representative live/dead staining images of dental plaque microcosm biofilms grown for two days on disks: (**A**) Commercial composite control; (**B**) RMGI; (**C**) Amalgam; and (**D**) Composite with 3% MPC. The composite control with 0% MPC had biofilms similar to (**A**) and was not included in Figure 2. Live bacteria were stained green, and dead bacteria were stained red.

Representative live/dead staining images of two-day biofilms grown on disks are shown in Figure 2: (A) Commercial composite control; (B) RMGI; (C) Amalgam; and (D) Composite with 3% MPC. The composite control with 0% MPC had biofilms similar to (A) and was not included in Figure 2. Composite with 3% MPC, RMGI, and Amalgam had noticeably less biofilm formation than commercial composite control.

**Figure 3:**
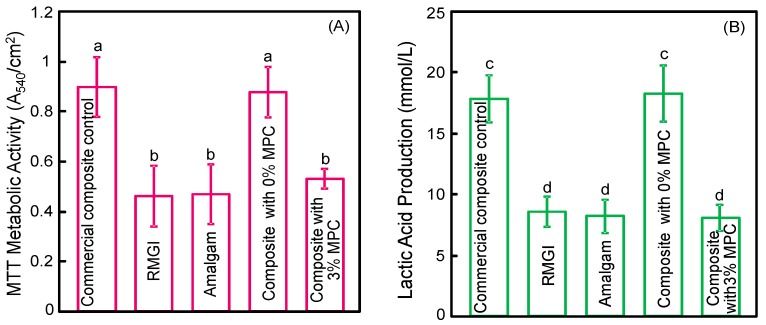
MTT metabolic assay and lactic acid production of biofilms on the surface of tested materials (mean ± sd; *n* = 6). Composite with 0% MPC and commercial composite control had biofilms with a relatively high metabolic activity and lactic acid production. However, adding 3% MPC decreased the metabolic activity and lactic acid by half. Values with dissimilar letters are significantly different from each other (*p* < 0.05). (**A**) MTT metabolic activity; (**B**) lactic acid production (mean ± sd; *n* = 6).

Quantitative biofilm viability results are plotted in Figure 3: (A) MTT metabolic activity; and (B) lactic acid production (mean ± sd; *n* = 6). Composite with 0% MPC and commercial composite had biofilms with a relatively high metabolic activity. Incorporation of 3% of MPC decreased the metabolic activity by half (*p* < 0.05), which became statistically similar to RMGI and Amalgam (*p* > 0.05). Lactic acid production by biofilms was similarly reduced for the composite containing 3% MPC, compared to the composite without MPC (*p* < 0.05).

**Figure 4:**
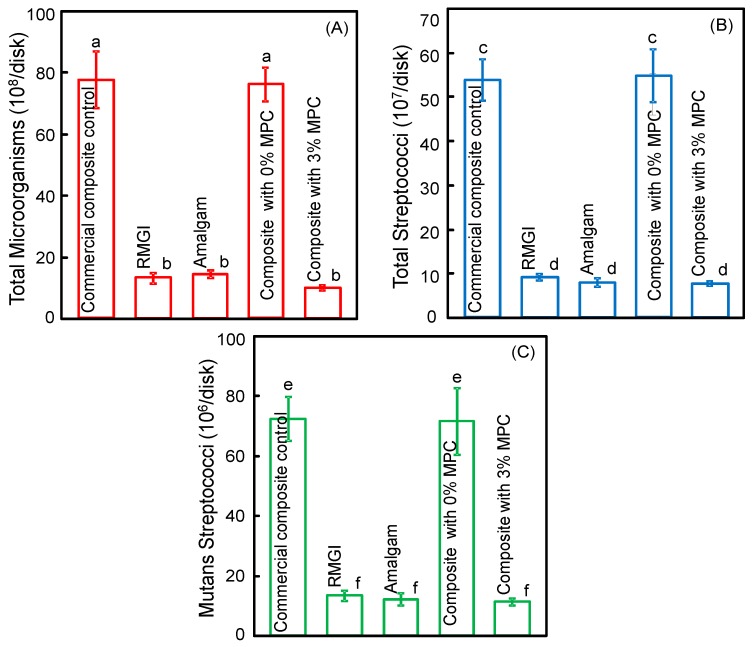
Colony-forming unit (CFU) counts of two-day biofilms on disks (mean ± sd; *n* = 6). (**A**) Total microorganism CFU; (**B**) total streptococci CFU; and (**C**) mutans streptococci CFU. In each plot, values with dissimilar letters are significantly different from each other (*p* < 0.05).

The two-day biofilm CFU counts are plotted in Figure 4: (A) Total microorganisms; (B) total streptococci; and (C) mutans streptococci (mean ± sd; *n* = 6). Composite with 3% MPC had less biofilm CFU than that with 0% MPC and commercial composite control (*p* < 0.05). The composite with 3% MPC had biofilm CFU similar to RMGI and Amalgam (*p* > 0.05).

**Figure 5:**
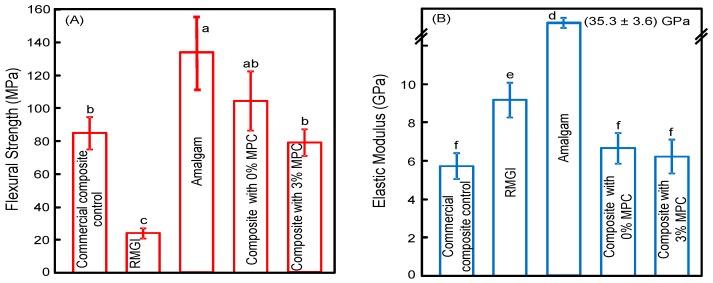
Flexural strength and elastic modulus of materials (mean ± sd; *n* = 6). Values with dissimilar letters are significantly different from each other (*p* < 0.05). (**A**) Flexural strength; (**B**) elastic modulus (mean ± sd; *n* = 6).

The mechanical properties are plotted in Figure 5: (A) Flexural strength; and (B) elastic modulus (mean ± sd; *n* = 6). The strength of the protein-repellent composite with 3% MPC matched those of the composite control with 0% MPC and the commercial composite control (*p* > 0.1). They all exceeded the strength of RMGI roughly four-fold. The elastic modulus of the protein-repellent composite with 3% MPC also matched those of the commercial composite and the control composite with 0% MPC (*p* > 0.1).

## 4. Discussion

Previous studied showed that dental composites accumulate more biofilms and plaques than amalgam and glass ionomer materials [36,37]; the present study demonstrated for the first time that a new protein-repellent composite reduced biofilm formation to be similar to those on amalgam and resin-modified glass ionomer. The hypotheses were proven that adding MPC into the composite substantially decreased the protein adsorption on the composite; that the new protein-repellent composite greatly reduced bacterial attachment, acid production, and biofilm CFU to match those of glass ionomer and amalgam materials which were known to deter biofilm growth; and that the new protein-repellent composite possessed mechanical properties similar to a commercial composite control which had no protein-repellent capability and which had much more bacterial attachment and lactic acid production.

There is a strong need to develop dental composites that can suppress biofilm formation, because recurrent caries around composite restorations have consistently been suggested as a primary reason for restoration failure [38]. Previous studies showed that the level of lactic acid-producing bacteria in the plaque on restoration surfaces is significantly higher on composite restorations than on amalgam and glass ionomer materials [36,37]. The methacrylate polymeric composition of resin composites permits attachment of salivary proteins and bacterial colonizers. Furthermore, it was suggested that the degradation products from common dental monomers such as BisGMA and TEGMA can alter the metabolism and promote the proliferation of *S. mutans* and biofilm formation [39]. Once biofilms are formed, the acid production by cariogenic bacteria can alter the composite surface roughness and porosity, which in turn can increase bacterial attachment and retention [40]. 

In the present study, indeed, the commercial composite control and the experimental composite without MPC had greater biofilm accumulation than amalgam and glass ionomer, consistent with previous reports [14,36,37]. The protein adsorption behavior for the commercial composite control and the experimental composite without MPC was similar to previous reports showing that dental resin composites had a relatively high adsorption capability, and consequently greater biofilm accumulation [41,42]. Indeed, dental resin composites did not show any anti-biofilm activity and had no caries-inhibitory effect [43].

In contrast, resin-modified glass ionomer and amalgam materials as direct-filling restorative materials could reduce biofilm accumulation. RMGI are a class of materials with fluoride-releasing capability that could provide a bacteriostatic effect [44]. Previous studies suggested that fluoride could significantly decrease the *S. mutans* levels in plaques by reducing the ability of *S. mutans* to ferment sucrose via a physicochemical mechanism [45]. However, the inferior mechanical properties of glass ionomers and resin-modified glass ionomers (e.g., the relatively lower strength in Figure 5A for RMGI) and the faster degradation and surface hardness loss compared to composites [45], have limited their use to non-load and low-load bearing tooth restoration areas. 

On the other hand, amalgam is a load-bearing direct-filling material. The bacteriostatic action of metallic elements such as silver ions released by amalgam into the biofilm is likely responsible for the observed lower biofilm formation. Indeed, previous studies showed the inhibition of *S. mutans* growth in planktonic cultures containing amalgam [46]. In addition, it was suggested that mercury (Hg) also contributed to the antimicrobial effects of amalgam [47]. Despite the reduced biofilm/plaque buildup and the low cost of amalgam, as well as its good marginal sealing and high longevity, patient demand for esthetic and metal-free restorations and the presence of mercury as an environmental hazard have led to a significant decrease of the use of amalgam [48]. These challenges indicate that the design and development of novel dental composites is urgently needed, as the antibacterial properties of composites may improve the restorative treatment outcome [49]. To date, there has been no report on a load-bearing and esthetic dental composite with low protein/bacteria attachment like amalgam and glass ionomer materials. 

This study showed that the composite with 3% MPC reduced the protein adsorption by an order of magnitude compared to commercial and experimental composite controls. The adhesion and metabolic activity of oral microorganisms were greatly reduced, so was lactic acid production, which was related to dental caries formation. The protein-repellent composite had mechanical properties similar to the commercial control composite which has been used clinically. MPC is a common biocompatible and hydrophilic biomedical polymer [20]. It is a methacrylate with a phospholipid polar group in the side chain, thus providing MPC with polymerization ability when used in a dental resin. Previous studies have confirmed the biocompatibility of MPC-containing materials and the ability to repel protein absorption, bacterial adhesion, and cellular attachment [20,50]. Regarding the protein-repellent mechanism, MPC is highly hydrophilic and there is an abundance of free water but no bound water in the hydrated MPC polymer. The presence of bound water would cause protein adsorption, while the large amount of free water around the phosphorylcholine group in MPC is considered to be responsible for detaching proteins, thereby repelling protein adsorption [20,50]. Indeed, several medical devices containing MPC have been approved by the Food and Drug Administration (FDA) and used clinically [51,52,53]. In the oral environment, protein adsorption from physiological fluids such as saliva-derived protein film on the surface is an initial required step and a pre-requisite for bacteria attachment [54]. Therefore, repelling proteins from the resin surface in the oral environment removed the attachment sites for bacteria, thereby making it more difficult for biofilm to grow on the resin. This would in turn help reduce biofilm acid production and secondary caries at the resin-tooth margins. The MPC-containing composite with greatly reduced biofilms suggests the potential application of MPC in dentistry, and a promising approach for addressing a major drawback of current dental composites which accumulate more biofilms than other restorative materials. While MPC is covalently bonded in the resin matrix and the protein-repellent function is expected to be long-term, further study is needed to investigate the duration to reduce biofilm formation, as well as the effects of different temperature conditions such as hot beverages and cold drinks on the properties of the protein-repellent composite. 

MPC-containing composites are expected to be useful in a wide range of applications including load-bearing restorations, both posterior and anterior cavity fillings, as well as flowable composites for root caries and Class V restorations to repel bacteria attachment and reduce biofilm acid production. Class V and Class II restorative margins may be subgingival, which could make cleaning difficult and provide areas and pockets for bacterial growth. It is generally accepted that overhanging restorations promote gingivitis by promoting the local accumulation of bacterial plaque, and restorations placed below the gingival margin could be detrimental to gingival and periodontal health [55,56,57]. For example, a previous study showed that over a one-year observation period of subgingival restorations, the composite group had a significant increase in the total bacterial counts, hence composite restorations may have some negative effects on the quantity and quality of subgingival plaque [56]. This could, in turn, lead to soft tissue inflammation, gingivitis, and the development of periodontitis in the area [55,56,57]. Therefore, the incorporation of MPC into a Class V or II restoration with gingival or subgingival margins could help repel proteins/bacterial attachment and growth, in order to reduce inflammation, gingivitis, and periodontitis in the area. Further studies are needed to investigate the effects of protein-repellent restorations on gingivitis and periodontitis. It should be noted that although MPC-containing resins can reduce bacterial adhesion, they have no bacteria-killing capability. Therefore, further studies are needed to incorporate both MPC and antibacterial agents into composites to possess double benefits of protein-repellent and bacteria-killing capabilities, to greatly reduce plaque buildup, secondary caries, as well as gingivitis and periodontitis.

## 5. Conclusions

The present study showed for the first time that a novel protein-repellent composite reduced oral biofilm growth matching amalgam and RMGI, while avoiding the non-esthetics of amalgam and the low strength of RMGI. Therefore, the long-held conclusion that dental resin composites accumulate more biofilms/plaque than amalgam and glass ionomer is no longer true. The new composite had mechanical properties comparable to those of the commercial composite control. The protein-repellent composite is promising for a wide range of dental applications to reduce biofilm acids and secondary caries, and the method of MPC incorporation may be applicable to other dental materials such as flowable composites, bonding agents, cements, and sealants.
